# Impact of Applying Magnetic Fields on the Development of Postbiotic Metabolites and Probiotic Microorganisms in Kombucha Tea

**DOI:** 10.1002/fsn3.70700

**Published:** 2025-07-25

**Authors:** Azize Atik, Gökhan Akarca, Ayşe Janseli Denizkara

**Affiliations:** ^1^ Food Technology Program Afyon Kocatepe University, Afyon Vocational School Afyonkarahisar Turkiye; ^2^ Faculty of Engineering, Department of Food Engineering Afyon Kocatepe University Afyonkarahisar Turkiye

**Keywords:** D‐glucuronic acid, magnetic field, postbiotics, SCOBY

## Abstract

The rising interest in fermented beverages is attributable to their content of probiotics and postbiotics. Kombucha, a fermented tea, captivates consumer interest. A natural source of probiotic bacteria and a variety of postbiotics, Kombucha is produced using a complex culture of different bacteria and yeasts (SCOBY). This study examined the physicochemical, microbiological, and textural changes of kombucha teas produced using SCOBY cultures subjected to varying magnetic field strengths (180 mT, 240 mT, and 300 mT) and durations (1, 2 h). The impact of magnetic field applications on the existence of probiotic bacteria and the generation of postbiotic metabolites in the samples was also examined. The total count of bacteria in SCOBY increased synchronously with the intensity and duration of the magnetic field. The highest *Lactobacillus* spp. bacteria and acetic acid bacteria and osmophilic yeast counts were determined in sample M2.2 with 4.08, 5.25, and 5.31 log cfu/mL, respectively, and the highest total yeast/mold count was determined in sample M3.2 with 5.15 log cfu/mL. The weight of the pellicle increased simultaneously with the increasing quantity of microorganisms. The maximum pellicle weight was recorded in the M2.2 sample at 101.91 g. The organic acid concentrations in the samples increased in parallel with the increase in microbial population. The maximum concentrations of acetic acid, D‐glucuronic acid, gluconic acid, lactic acid, and citric acid were 6.30, 1.65, 3.28, 0.38, and 0.054 mg/100 mL in the M2.2 sample, respectively. Furthermore, applying the magnetic field at the end of fermentation resulted in elevated levels of total phenolic compounds and enhanced antioxidant capacity. The significance of magnetic field applications in improving the health and nutritional attributes of fermented beverages like Kombucha tea, which have gained consumer preference in recent years, by augmenting D‐glucuronic acid and postbiotic metabolites has been unequivocally established.

## Graphical abstract

The process supported the growth of bacteria and yeasts, forming SCOBY culture. The amount of D‐glucuronic acid increased at the end of fermentation. Kombucha tea is enriched with probiotic microorganisms and postbiotic metabolites.

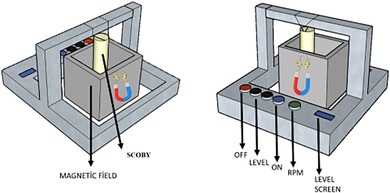

## 1 Introduction

Kombucha, a fermented tea, has garnered the interest of health‐conscious persons since ancient times. This distinctive beverage, created by the symbiotic fermentation of tea, sugar, and a specific culture, has garnered considerable interest among the scientific community owing to its prospective health advantages and rising market demand (Dufresne and Farnworth 2000; da Silva Júnior et al. 2022). Basically, a complex microbial population of yeasts and acetic acid bacteria works in tandem to produce kombucha through a symbiotic fermentation process (Al‐Mohammadi et al. 2021).

The kombucha market has demonstrated notable growth. The data indicates an increase in value from USD 1.84 billion in 2019 to around USD 2.64 billion by 2021. Predictions indicate sustained robust growth, with the market anticipated to attain a value between US$10.26 billion and US$10.45 billion by 2027, reflecting a compound annual growth rate (CAGR) of around 15.6%–24.8% throughout this timeframe (Estrada et al. 2023). An emerging trend is the diversification of flavors, with brands presenting increasingly inventive mixes of fruits and herbs to attract a broader audience (Wang et al. 2022).

The kombucha fermentation process commences with the formulation of a sweetened black or green tea solution, which is inoculated with a microbial culture referred to as a “SCOBY”. The SCOBY, or “Symbiotic Culture of Bacteria and Yeasts,” is a gelatinous, cellulose membrane that serves as a starter for fermentation (da Silva Júnior et al. 2022). During fermentation, typically lasting between 7 and 30 days, SCOBY metabolizes the tea's sugars to generate various bioactive compounds, including organic acids, polyphenols, and vitamins (Al‐Mohammadi et al. 2021).

The microbial community in kombucha is varied and includes more than 200 species, featuring yeasts like *Saccharomyces cerevisiae* , *Zygosaccharomyces bailii*, and *Schizosaccharomyces pombe*, along with Acetic Acid Bacteria (AAB) such as *Acetobacter xylinum* and *Gluconobacter* spp. (Lee et al. 2021; Herwin 2022; Wang et al. 2022). The fermentation process starts with transforming sucrose into ethanol by yeasts, succeeded by the oxidation of ethanol to acetic acid by acetic acid bacteria, culminating in the distinctive sour flavor of kombucha (Yuliana and Permana 2022; Antolak et al. 2021). The equilibrium among these bacteria is critical as it affects the sensory and health‐enhancing attributes of the final product (Kaashyap et al. 2021; Yang 2023). Kombucha is regarded as a probiotic beverage because of the variety of naturally existing symbiotic microbes. The health benefits of kombucha arise from short‐chain fatty acids and other metabolites produced during fermentation, which enhance immunity and provide antiviral, antibacterial, and antifungal effects (Nyiew et al. 2022).

The application of static or moving magnetic fields influences microbial growth. The magnetic field induces alterations in the DNA synthesis of microorganisms, influences the arrangement of biomembranes or biomolecules, and affects the ionic movement across the plasma membrane, ultimately impacting the cell's proliferation rate (Açu et al. 2014). The strength and duration of the applied magnetic field influence microorganisms. The impact of SCOBY culture subjected to magnetic field application on Kombucha fermentation was examined in this study based on this premise.

The stability and diversity of microbial populations may vary markedly between many fermentation batches, influencing the flavor and health benefits of the final product (Mas et al. 2022; Landis et al. 2022). The presence of lactic acid bacteria (LAB) in SCOBY, in addition to acetic acid bacteria (AAB) and yeasts, indicates a complex interaction that could potentially enhance its probiotic properties (Wang et al. 2022; Antolak et al. 2021). Using a magnetic field on SCOBY has induced alterations in the bacteria and yeasts; therefore, it influences the fermentation process and, consequently, the physicochemical and sensory characteristics of the final product.

Postbiotics, belonging to the biotics family, are bioactive components generated by microorganisms throughout the fermentation process (Wegh et al. 2019). Postbiotics are present in functional and enriched foods, including probiotics and prebiotics (Birch and Bonwick 2019; Rad et al. 2020). Postbiotics are defined as any substance generated by probiotic fermentation that confers health advantages to the host (Ji et al. 2023). This concept expands the scope from viable microorganisms to encompass many metabolites, including polysaccharides, peptides, and short‐chain fatty acids (SCFAs) (Aggarwal et al. 2022). Postbiotics, known as metabiotics, biogenics, or metabolites, include soluble factors (products or metabolic by‐products) secreted by living bacteria during fermentation or after bacterial lysate. Soluble factors include short‐chain fatty acids, enzymes, peptides, teichoic acids, muropeptides derived from peptidoglycan, endo‐ and exopolysaccharides, cell surface proteins, vitamins, plasmalogens, pilus‐type structures, and organic acids (Cicenia et al. 2014; Aguilar‐Toalá et al. 2018).

The influence of magnetic fields on microorganism growth is well‐documented. Reports indicate that magnetic fields enhance growth rates and biomass accumulation across various species (Vanags et al. 2010). Furthermore, it was highlighted that magnetically assisted applications can substantially control microbial growth and enhance the production of secondary metabolites, which possess important utility in biotechnological processes (Jabłońska et al. 2022).

This study investigated the alterations in physicochemical, microbiological, and textural properties of kombucha teas produced with SCOBY cultures subjected to varying intensities and durations of magnetic field exposure. Furthermore, the impact of magnetic field applications on probiotic microorganisms and postbiotic metabolite formation in kombucha samples was examined.

## 2 Materials and Methods

### 2.1 Research Design

**FIGURE 1:**
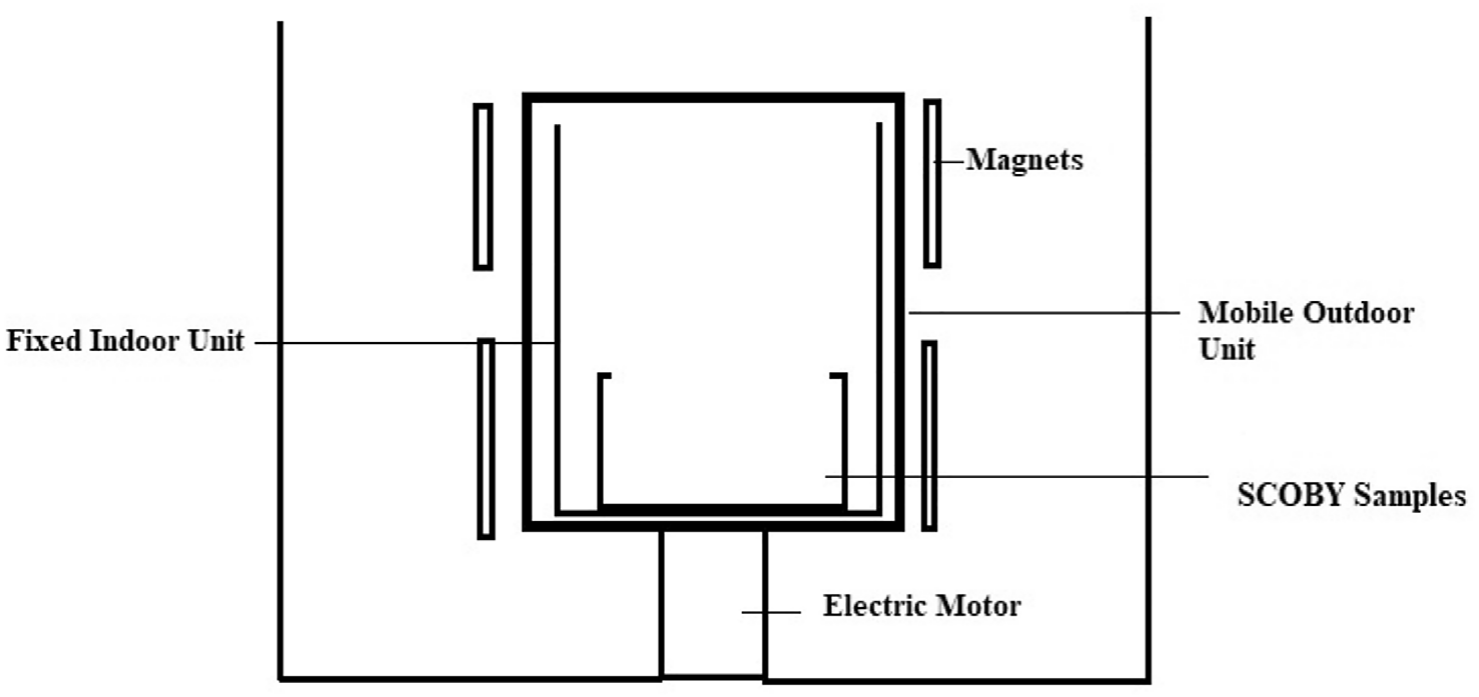
The magnetic field mechanism used in the research.

The research design was specifically created for this purpose, utilizing aluminium material (Figure 1). The device's rotating component was adjustable through the use of a rotor. Within the interior, an immobile section was where the kombucha pellicles were situated. The rotating component was designed as a square prism, featuring 12 interchangeable magnets (3 on each side) and was linked to a rotor with adjustable rotation speed. The rotor speed was kept constant in the study and set to 80 rpm. After each experiment, the magnets were changed, and magnetic fields of three different strengths were obtained. The strength of the magnets was determined as 15, 20, and 25 mT. Accordingly, a magnetic field with a total power of 180, 240, and 300 mT was created in the application. The intensity of the applied magnetic field was determined through preliminary experiments, utilizing intensities that would not adversely affect the microorganisms present in SCOBY. Initial experiments indicated that applying magnetic fields at intensities exceeding 300 mT inactivated microorganisms. Consequently, the maximum intensity of the magnetic field was set at 300 mT for applications. During the research, the strength of the magnetic field was measured continuously with the help of a tesla metre. The magnetic field strength was measured in the range of 168–199 mT for the 1st trial, 244–263 mT for the 2nd trial, and 271–321 mT for the 3rd trial.

### 2.2 Production of Kombucha Tea

Kombucha teas were produced per the parameters outlined in Akarca (2021). 25 g of black tea (*Camelia sinensis*) were measured using a precision balance, and 1000 mL of potable water was included. Sucrose (100 g/L) was added to the mixture, which was then stirred at 95°C for 20 min to facilitate melting. The mixture was infused for 20 min after the methods were implemented. The solution was filtered using sterile filter paper (Whatman No. 32). The mixture was then placed in sealed jars and sterilized in an autoclave (Nüve‐OT 90 L, Turkey) at 121°C for 20 min. The mixture was cooled to 25°C. Then, 45 g/L of the pellicle phase (both control and treatment) and 150 mL of the liquid phase of the pre‐produced Kombucha tea were added to the mixture. The samples were left to ferment in a dark environment at 24°C ± 3°C for 21 days.

### 2.3 Proximate Analyses

The percentage of titratable acidity (m/m acetic acid equivalent) and Brix (% soluble dry matter content) of Kombucha tea samples was assessed using a benchtop refractometer (Atago Rx 5000, Japan), following the methodologies outlined by Yıkmış and Tuğgüm (2019) and Ayed et al. (2017), respectively. Color values (L*, a*, and b*) were determined using colourimetry (Konika Minolta Chromium Meter CR‐400) as per Abuduaibifu and Tamer (2019), while pellicle weights were measured using a precision balance (Radwag PS‐1000 R2, Poland).

### 2.4 Total Antioxidant Capacity

DPPH• (2,2‐diphenyl‐1‐picrylhydrazyl) solution was prepared at a concentration of 60 μ/mL. 3.9 mL of DPPH solution was first added to the test tubes, and then 100 μL of samples were added to these tubes. The tubes were incubated for 30 min at room temperature in the dark. After 30 min of incubation, the absorbance was measured at 517 nm against the ethanol blank. A 60 μL DPPH• solution was used as a control solution. Decreasing absorbance gives the remaining amount of DPPH• solution. This shows the free radical scavenging activity.

The amount of DPPH• removed from the solution was determined using the subsequent equation.

$$\text{DPPH}•\text{Reduction Percentage}\;\left(\%\right)=\left[\left(\text{A0}–A1\right)/\text{A0}\right]\times 100$$

A0: Absorbance of the control,

A1: Absorbance of the samples (Velićanski et al. 2014).

### 2.5 Total Phenolic Content

The total phenolic content of the samples was determined using the Folin–Ciocalteu colorimetric method. Based on their composition, the samples were diluted to ratios of 1/50, 1/200, and 1/300 for analysis. 0.5 mL of diluted sample, 2 mL of 1/10 diluted Folin, and 1 mL of 7% Na_2_CO_3_ were combined and vortexed. The samples were kept in a water bath at 40°C for 30 min. Absorbances were subsequently measured using a spectrophotometer at a wavelength of 760 nm. Gallic acid served as the standard. The results were documented as mg of gallic acid equivalent (GAE) per gram of dry weight (Velićanski et al. 2014).

### 2.6 Texture Analysis

The tea samples' texture parameters (firmness, consistency, cohesiveness, and viscosity index) were determined using a TA.XT Plus Texture Analyser (Stable Micro Systems, Godalming, Surrey, UK) with back extrusion rig apparatus (TA‐30A, 7.62 cm diameter, cylindrical acrylic, and 10 mm height) (Akarca and Denizkara 2024).

### 2.7 Organic Acid Analysis

The organic acid concentration in the samples was quantified by HPLC (Shimadzu Prominence, Shimadzu Corporation, Kyoto, Japan). 4 g of the sample were weighed, 20 mL of 0.01 N H_2_SO_4_ was added, the mixture was vortexed, and subsequently fed into the system via a 0.45 μm filter (Güzel‐Seydim et al. 2000). System properties used: CBM: 20ACBM; detector: DAD (SPD‐M20A); column oven: CTO‐10ASVp; pump: LC20 AT; autosampler: SIL 20ACHT; computer program: LC solution; column: ODS 4 (250 mm × 4.6 mm, 5 μm; Inertsil ODS‐4, GP Sciences, Nakatsugawa, Japan); mobile phase: ultrapure water adjusted to pH 3 with orthophosphoric acid. In the mobile phase, a flow rate of 0.7 mL min^−1^ and detection at a wavelength of 210 nm were used (Hakan et al. 2005).

### 2.8 Microbiological Analyses

Kombucha samples of 1 mL each were taken from the prepared dilutions using a sterile automatic pipette (Research Plus, Eppendorf, Germany) and inoculated into media appropriate for microbiological analysis, subsequently spread uniformly with a sterile Drigalski spatula (Fıratpen, Turkey) following the spreading plate method. de Man, Rogosa and Sharpe (MRS) Agar (Merck, Germany) was utilized to analyze *Lactobacillus* spp. bacteria count, with incubation conducted at 30°C under anaerobic conditions for 24–48 h. For the analysis of acetic acid bacteria counts, Yeast Extract Calcium Carbonate Glucose (YCG) Agar (Himedia, India) was utilized, with incubation conducted at 30°C under aerobic conditions for 5–10 days (Neffe‐Skocińska et al. 2017). Rose Bengal Chloramphenicol Agar (Merck, Germany) was utilized for total yeast and mold count analysis, incubated at 22°C for 3–5 days under aerobic conditions (ISO 2008a). Osmophilic yeast count analysis was conducted using DG‐18 agar (Merck, Germany), with incubation occurring for 5–7 days at 30°C under aerobic conditions (ISO 2008b).

### 2.9 Sensory Analysis

A sensory evaluation of Kombucha teas was carried out at the end of the fermentation. Scorecards created by modifying the sensory test parameters specified by Malbasa et al. (2014) were used in these analyses. The analyses were conducted by 20 panelists, comprising academic members and PhD and MSc students. Samples were evaluated with a 1–8 hedonic scale in terms of taste, structure, color, acidity, odor, and general acceptability.

### 2.10 Statistical Analyses

In this investigation, a two‐way analysis of variance was utilized to assess the differences (*p* < 0.05) between the samples using doubling parallel and doubling replicate. The interactions between applied magnetic field intensity, application time, and magnetic field intensity × application time were determined using correlation analysis. The analysis findings were subjected to the ANOVA process followed by Duncan's multiple range tests (SPSS, version 28). The design was entirely randomized with replications.

## 3 Results and Discussion

The results for titratable acidity, brix, antioxidant activity (DPPH), total phenolic content, and pellicle weight of the samples are shown in Table 1. According to the results of the variation analysis, it was determined that magnetic field intensity interaction was very highly significant (*p* < 0.0001) on the DPPH value and the pellicle weight. In addition, time interaction was very highly significant (*p* < 0.0001) on the brix value and highly significant (*p* < 0.01) on the pellicle weight. Furthermore, magnetic field intensity × time interaction was very highly significant (*p* < 0.0001) on titratable acidity, the DPPH value, and the pellicle weight. In the results of the correlation analysis, it was revealed that magnetic field intensity interaction had a very highly negative correlative effect on the brix value.

**TABLE 1:** Change of titratable acidity (%), brix (%), antioxidant activity (%), total phenolic content (TPC), and pellicule weight (g) on Kombucha samples.

Samples	Titratable acidity %	Brix (%)	DPPH (%)	Total phenolic content (mg GAE/g)	Pellicule weight (g)
Control	12.99 ± 0.75^c^	9.00 ± 0.32^a^	59.95 ± 1.90^c^	208.83 ± 3.49^c^	65.40 ± 2.76^d^
M1.1	13.60 ± 0.67b^c^	8.36 ± 0.35^ab^	62.99 ± 0.27^b^	220.27 ± 4.04^ab^	77.05 ± 3.99^c^
M2.1	14.93 ± 0.55^b^	8.14 ± 0.52^ab^	64.36 ± 1.01^b^	219.36 ± 1.04^ab^	83.74 ± 2.01^b^
M3.1	12.82 ± 0.43^c^	8.90 ± 0.29^a^	58.75 ± 0.50^c^	221.49 ± 4.30^ab^	66.76 ± 2.67^d^
M1.2	14.62 ± 0.57^b^	6.66 ± 0.54^cd^	63.72 ± 0.66^b^	216.33 ± 1.41^b^	76.68 ± 2.75^c^
M2.2	17.64 ± 1.08^a^	5.79 ± 0.65^d^	67.53 ± 0.89^a^	223.73 ± 1.12^a^	101.91 ± 2.76^a^
M3.2	11.97 ± 0.13^c^	7.34 ± 0.19^bc^	57.82 ± 0.55^c^	215.90 ± 2.19^b^	64.62 ± 2.23^d^
Variation (*p* value)					
M	0.007	0.121	< 0.0001	0.008	< 0.0001
T	0.049	< 0.0001	0.324	0.170	0.010
M × T	< 0.0001	0.047	< 0.0001	0.075	< 0.0001
Correlation (*r*)					
M	−0.249	−0.861**	−0.351	0.479	0.611
T	0.319	0.259	0.228	0.116	0.311

*Note:* M1.1, 180mT 1 h; M2.1, 240mT 1 h; M3.1, 300mT 1 h; M1.2, 180mT 2 h; M2.2, 240mT 2 h; M3.2, 300mT 2 h. Values with the different letters in the same column for each analysis differ significantly (*p* < 0.05). Statistical significance: *p* < 0.0001, very highly significant; *p* < 0.01, highly significant; *p* < 0.05, significant; *p* > 0.05, not significant.

Abbreviations: M, magnetic field intensity; T, time (hour).

** Correlation significance is indicated by asterisks: Very highly correlative effect (2‐tailed).

Titratable acidity, an indicator of total acidity in foods, was determined in terms of acetic acid in the samples. The magnetic field applied to the samples showed an increasing effect on the titratable acidity values of the samples (*p* < 0.05). Although the magnetic field intensity showed an increasing effect up to 200 mT, the value of the effect occurred in magnetic field applications above this value increased. In addition, the increase detected in the titratable acidity values of the samples increased depending on the applied time (*p* < 0.05). Among the samples, the highest increase in titratable acidity value was found in the M2.2 sample with a value of 17.64% and the lowest increase was found in the control sample with a value of 12.99%. The increase in titratable acidity depends on the count of acetic acid bacteria (AAB) and the amount of acetic acid, which increases with the applied magnetic field intensity and duration. On the other hand, the brix value decreased depending on the applied magnetic field intensity (*p* < 0.05). The highest brix value of 9% was determined in the control sample, and the lowest brix value of 5.79% was determined in the M2.2 sample. The decrease in brix value was due to the hydrolysis of sucrose to glucose and fructose by yeasts during fermentation and the conversion of glucose and fructose to ethanol by acetic acid bacteria through glycolysis (Akarca and Tomar 2020). In general, magnetic field application increased the antioxidant activity of the samples. DPPH value increased depending on magnetic field intensity and duration. It was observed that the DPPH value decreased only in the M3.2 sample, and the lowest value, 57.8,2%, was measured in this sample. The highest DPPH value was 67.53% in the M2.2 sample. Total phenolic content increased with magnetic field application (*p* < 0.05). The highest total phenolic content was 223.73 mg GAE/g in the M2.2 sample. Magnetic field applications had an increasing effect on pellicle biomass weight (*p* < 0.05). The pellicle, often referred to as the “mother” or “fungus”, is a gelatinous, cellulosic film that forms on the surface of kombucha tea during the fermentation process (Wang et al. 2023). This unique structure comprises a symbiotic community of bacteria and yeast that work together to transform the sweetened tea into the tangy, bubbling beverage we know as kombucha. The pellicle is thought to consist of a network of cellulose fibers that act as a scaffold for the diverse microbial community. Microorganisms within the pellicle, including acetic acid bacteria and yeasts, work together to produce a variety of compounds such as organic acids, polyphenols and even potentially antimicrobial substances (Sreeramulu et al. 2001; Nyiew et al. 2022). Considering the results of microbiological analysis, in general, magnetic field application caused an increase in the count of SCOBY‐forming bacteria and yeasts (*p* < 0.05). It is thought that the pellicle weight increased in parallel with this increase. The lowest pellicle weight was determined in the M3.2 sample with 64.62 g, and the highest pellicle weight was determined in the M2.2 sample with 101.91 g.

The results for the textural properties of the samples are given in Table 2. According to the results of the variation analysis, it was determined that magnetic field intensity × time interaction was very highly significant (*p* < 0.0001) on the firmness value. On cohesiveness and index of viscosity, time and magnetic field intensity × time interactions were highly significant (*p* < 0.01), while on consistency, magnetic field intensity, time, and magnetic field intensity × time interactions were significant (*p* < 0.05). In the results of the correlation analysis, it was revealed that magnetic field intensity interaction had a highly positive correlative effect (*r* = 0.643) on the cohesiveness value. The cohesiveness value increased depending on magnetic field intensity and application time. The lowest value was measured in the control sample (−4.29), and the highest was in the M2.2 sample (−3.76). Magnetic field application had no significant effect on the consistency value. Magnetic field application caused a slight increase in firmness value. The index of viscosity value increased with magnetic field application. Although the precise mechanisms via which magnetic field application influences Kombucha's textural characteristics remain unclear, several theories have been put out in the literature to date (Sreeramulu et al. 2001; Massoud et al. 2022). According to one theory, applying the magnetic field could impact the development and metabolic activity of the microbial consortia in charge of the fermentation process, which might change how organic acids and other metabolites are produced. Furthermore, the application of magnetic field treatment has the potential to influence the physicochemical properties of Kombucha. This includes alterations in the solubility and interactions of the various compounds found within the beverage, ultimately leading to modifications in the overall texture and mouthfeel of the final product (Sreeramulu et al. 2001). Additionally, the application of magnetic field treatment has the potential to modify the physicochemical properties of Kombucha directly. This includes alterations in the solubility and interactions of different compounds, which may subsequently impact the final product's viscosity, mouthfeel, and overall textural profile. The observed effect on physicochemical properties likely stems from the magnetic field's capacity to influence the solubility and intermolecular interactions among various compounds found in Kombucha, including organic acids, polyphenols, and other secondary metabolites. The alterations in solubility and compound interactions could result in variations in viscosity, mouthfeel, and the overall structural characteristics of Kombucha tea (Massoud et al. 2022).

**TABLE 2:** Change of textural properties of Kombucha samples.

Samples	Cohesiveness	Consistency (gs)	Firmness (g)	Index of viscosity (gs)
Control	−4.29 ± 0.10^d^	65.29 ± 0.03^ab^	10.68 ± 0.04^c^	−0.64 ± 0.01^e^
M1.1	−4.06 ± 0.06^bc^	65.34 ± 0.06^a^	10.79 ± 0.04^b^	−0.61 ± 0.01^cde^
M2.1	−3.99 ± 0.04^bc^	65.37 ± 0.06^a^	10.92 ± 0.04^a^	−0.59 ± 0.01^bc^
M3.1	−4.19 ± 0.01^cd^	65.27 ± 0.02^ab^	10.74 ± 0.05^bc^	−0.60 ± 0.01^cd^
M1.2	−3.86 ± 0.06^b^	65.34 ± 0.05^a^	10.78 ± 0.04^b^	−0.56 ± 0.01^ab^
M2.2	−3.76 ± 0.15^a^	65.39 ± 0.04^a^	10.95 ± 0.04^a^	−0.53 ± 0.01^a^
M3.2	−4.08 ± 0.08^c^	65.19 ± 0.05^b^	10.53 ± 0.03^d^	−0.63 ± 0.01^de^
Variation (*p* value)				
M	0.090	0.100	0.002	0.021
T	0.003	0.384	0.088	0.003
M × T	0.008	0.017	< 0.0001	0.001
Correlation (*r*)				
M	0.643*	−0.298	−0.079	0.697
T	−0.182	−0.070	−0.108	0.476

*Note:* M1.1, 180 mT 1 h; M2.1, 240 mT 1 h; M3.1, 300 mT 1 h; M1.2, 180 mT 2 h; M2.2, 240 mT 2 h; M3.2, 300 mT 2 h. Values with the different letters in the same column for each analysis differ significantly (*p* < 0.05). Statistical significance: *p* < 0.0001, very highly significant; *p* < 0.01, highly significant; *p* < 0.05, significant; *p* > 0.05, not significant.

Abbreviations: M, magnetic field intensity; T, time (hour).

* Correlation significance is indicated by asterisks: highly correlative effect (2‐tailed).

The microorganism counts for the samples are shown in Table 3. The magnetic field intensity and the application time influenced microbial growth. According to the results of the variation analysis, it was determined that the magnetic field intensity × time interaction was very highly significant (*p* < 0.0001) on all microorganism groups analyzed. In addition, it was observed that the magnetic field intensity was very highly significant (*p* < 0.0001) on yeast/mold and osmophilic yeast growth. The magnetic field intensity was highly significant (*p* < 0.01) on the *Lactobacillus* spp. bacteria and AAB growth. The magnetic field application caused a significant increase in *Lactobacillus* spp. bacteria, AAB, and osmophilic yeast counts in general (*p* < 0.05). However, as the applied magnetic field intensity and duration increased, it was observed that the count of microorganisms decreased again. The lowest *Lactobacillus* spp. bacteria, AAB, and osmophilic yeast counts were 2.74, 3.98, and 4.34, respectively, in sample M3.2. On the other hand, the magnetic field application caused a decrease in yeast/mold counts at first but increased again depending on the intensity and duration of the application. The lowest yeast/mold count was determined in the M2.2 sample (4.29 cfu/mL), and the highest yeast/mold count was determined in the M3.2 sample (5.15 cfu/mL).

**TABLE 3:** Microbial count of Kombucha samples (log CFU/mL).

Samples	*Lactobacillus* spp. bacteria	Acetic acid bacteria	Yeast/Mold	Osmophilic yeast
Control	2.99 ± 0.17^c^	4.00 ± 0.16^e^	5.12 ± 0.06^a^	4.45 ± 0.03^cd^
M1.1	3.18 ± 0.04^c^	4.39 ± 0.09^cd^	4.98 ± 0.16^ab^	4.58 ± 0.11^c^
M2.1	3.73 ± 0.07^b^	4.96 ± 0.13^b^	4.82 ± 0.08^b^	4.92 ± 0.08^b^
M3.1	3.09 ± 0.07^c^	4.19 ± 0.05^de^	5.07 ± 0.06^a^	4.48 ± 0.04^cd^
M1.2	3.57 ± 0.13^b^	4.59 ± 0.09^c^	5.04 ± 0.07^a^	4.61 ± 0.03^c^
M2.2	4.08 ± 0.06^a^	5.25 ± 0.11^a^	4.29 ± 0.05^c^	5.31 ± 0.06^a^
M3.2	2.74 ± 0.10^d^	3.98 ± 0.16^e^	5.15 ± 0.06^a^	4.34 ± 0.11^e^
Variation (*p* value)				
M	0.003	0.002	< 0.0001	< 0.0001
T	0.028	0.055	0.019	0.046
M × T	< 0.0001	< 0.0001	< 0.0001	< 0.0001
Correlation (*r*)				
M	−0.187	−0.070	−0.298	−0.227
T	0.244	0.241	0.046	0.046

*Note:* M1.1, 180 mT 1 h; M2.1, 240 mT 1 h; M3.1, 300 mT 1 h; M1.2, 180 mT 2 h; M2.2, 240 mT 2 h; M3.2, 300 mT 2 h. Values with the different letters in the same column for each analysis differ significantly (*p* < 0.05). Statistical significance: *p* < 0.0001, very highly significant; *p* < 0.01, highly significant; *p* < 0.05, significant; *p* > 0.05, not significant.

Abbreviations: M, magnetic field intensity; T, time (hour).

Kombucha tea is a highly efficient natural probiotic source (Altunatmaz et al. 2010). Using magnetic fields augmented the concentration of probiotic bacteria in kombucha tea samples. Exposure to a magnetic field diminishes the viability of microorganisms. However, this effect varies depending on the intensity of the exposed field, duration, and the species/strain of the microorganism. The magnetic field produces biomolecular and chemical effects (affecting the electronic spin states of reaction intermediates) in the cell cytoplasm of microorganisms, mainly lactic acid bacteria. The emerging effect may induce alterations in intracellular ion homeostasis (Pei et al. 2007), enzyme activities (Dang et al. 2007), cell shape, cell development (Bochu et al. 2002), and cell division (Naruse 2002).

Research indicates that most biological tissues exhibit diamagnetic properties (Liu et al. 2017). The diamagnetic response to an external magnetic field induces magnetic induction in the opposite direction (Butler 2014). A low‐intensity, low‐frequency magnetic field influences ion movement across the cell membrane (Wang and Hladky 1994). Furthermore, the magnetic field influences the conductance of K^+^ channels in the cell membrane (Cecchetto et al. 2012). The alterations in cell membrane conductivity subsequently influence microbial growth. Minor alterations promote microbial growth, but higher and substantial alterations impede growth (Tirono et al. 2018).

The organic acid amounts of the samples are given in Table 4. The magnetic field intensity and the application time influenced the organic acid amounts formed during the fermentation process. According to the results of the variation analysis, it was determined that magnetic field intensity and magnetic field intensity × time interactions were very highly significant (*p* < 0.0001) on acetic acid and gluconic acid, while magnetic field intensity × time interaction was very highly significant (*p* < 0.0001) on D‐glucuronic acid, lactic acid, and citric acid. In addition, magnetic field intensity interaction was highly significant (*p* < 0.01) on D‐glucuronic acid, lactic acid, and citric acid, while time interaction was highly significant (*p* < 0.01) on D‐glucuronic acid and gluconic acid. Acetic acid and D‐glucuronic acid content increased with magnetic field application (*p* < 0.05). The amounts of gluconic acid, lactic acid, and citric acid generally increased but decreased depending on the magnetic field intensity and application time. The highest amount for all organic acids was determined in the M2.2 sample. The lowest amounts were determined in the control sample for acetic acid and D‐glucuronic acid (4.76 and 1.40, respectively) and in the M3.2 sample for gluconic acid, lactic acid, and citric acid (2.48, 0.14, and 0.028, respectively).

**TABLE 4:** Organic acids of Kombucha samples (mg/100 mL).

Samples	Acetic acid	D‐glucuronic acid	Gluconic acid	Lactic acid	Citric acid
Control	4.76 ± 0.14^d^	1.40 ± 0.01^d^	2.70 ± 0.06^cd^	0.19 ± 0.02^c^	0.031 ± 0.005^c^
M1.1	5.02 ± 0.08^cd^	1.46 ± 0.03^c^	2.71 ± 0.03^cd^	0.23 ± 0.02^bc^	0.039 ± 0.003^c^
M2.1	5.96 ± 0.12^b^	1.56 ± 0.01^b^	2.76 ± 0.04^bc^	0.28 ± 0.02^b^	0.043 ± 0.001^b^
M3.1	4.95 ± 0.13^cd^	1.44 ± 0.02^cd^	2.59 ± 0.04^de^	0.20 ± 0.02^c^	0.031 ± 0.002^c^
M1.2	5.08 ± 0.06^c^	1.52 ± 0.02^b^	2.87 ± 0.08^b^	0.24 ± 0.02^bc^	0.043 ± 0.003^b^
M2.2	6.30 ± 0.11^a^	1.65 ± 0.03^a^	3.28 ± 0.05^a^	0.38 ± 0.03^a^	0.054 ± 0.002^a^
M3.2	4.81 ± 0.09^cd^	1.43 ± 0.01^cd^	2.48 ± 0.09^e^	0.14 ± 0.01^d^	0.028 ± 0.004^c^
Variation (*p* value)					
M	< 0.0001	0.002	< 0.0001	0.001	0.004
T	0.049	0.002	0.003	0.235	0.052
M × T	< 0.0001	< 0.0001	< 0.0001	< 0.0001	< 0.0001
Correlation (*r*)					
M	0.809	−0.068	−0.390	−0.153	−0.227
T	0.195	0.405	0.384	0.168	0.332

*Note:* M1.1, 180 mT 1 h; M2.1, 240 mT 1 h; M3.1, 300 mT 1 h; M1.2, 180 mT 2 h; M2.2, 240 mT 2 h; M3.2, 300 mT 2 h. Values with the different letters in the same column for each analysis differ significantly (*p* < 0.05). Statistical significance: *p* < 0.0001, very highly significant; *p* < 0.01, highly significant; *p* < 0.05, significant; *p* > 0.05, not significant.

Abbreviations: M, magnetic field intensity; T, time (hour).

Kombucha tea is highly abundant in glucuronic acid. The antioxidative action of tea correlates directly with the ratio of D‐glucuronic acid. Furthermore, D‐glucuronic acid enhances the host's absorption of polyphenols (Dufresne and Farnworth 2000; Teoh et al. 2004). The concentration of D‐glucuronic acid increases during kombucha fermentation (Dufresne and Farnworth 2000). The use of magnetic fields markedly elevated the D‐glucuronic acid concentration in teas.

The microbial population within Kombucha SCOBY constitutes a rich and dynamic ecosystem characterized by a delicate equilibrium of acetic acid bacteria and yeasts (Harrison and Curtin 2021). Acetic acid bacteria, including Acetobacter and Gluconobacter, are the predominant microorganisms in SCOBY and are responsible for synthesizing acetic acid, the principal flavor compound in Kombucha. These bacteria are crucial in fermentation, using glucose to generate gluconic acid and ethanol to yield acetic acid (Soares et al. 2021). The microbial population can produce other organic acids, like gluconic acid and lactic acid, alongside acetic acid, enhancing the overall complexity of Kombucha's flavor profile (van Wyk et al. 2023).

One of the primary metabolic steps in Kombucha fermentation is the transformation of ethanol into acetic acid by acetic acid bacteria, including Acetobacter and Gluconobacter (Sreeramulu et al. 2001; Abaci et al. 2022). A significant metabolic activity in kombucha fermentation is gluconic acid synthesis by some acetic acid bacteria, including Gluconobacter. Magnetic fields can accelerate microorganism growth (Masood 2017). For this reason, a magnetic field may accelerate the fermentation process (Sánchez‐Clemente et al. 2018). Prior research indicates that using low doses of magnetic fields positively influences the growth of microorganisms, mainly lactic acid bacteria (Segatore et al. 2012). However, the growth of microorganisms is slowed down or even stopped by large dosages and prolonged magnetic field treatments (Peña Guzmán et al. 2019). The microbiological counting findings of the samples show a comparable relationship with the change in the quantity of organic acids. It was found that the quantity of related organic acids rose in the samples with more bacteria, contingent on the duration and intensity of the magnetic field.

**FIGURE 2:**
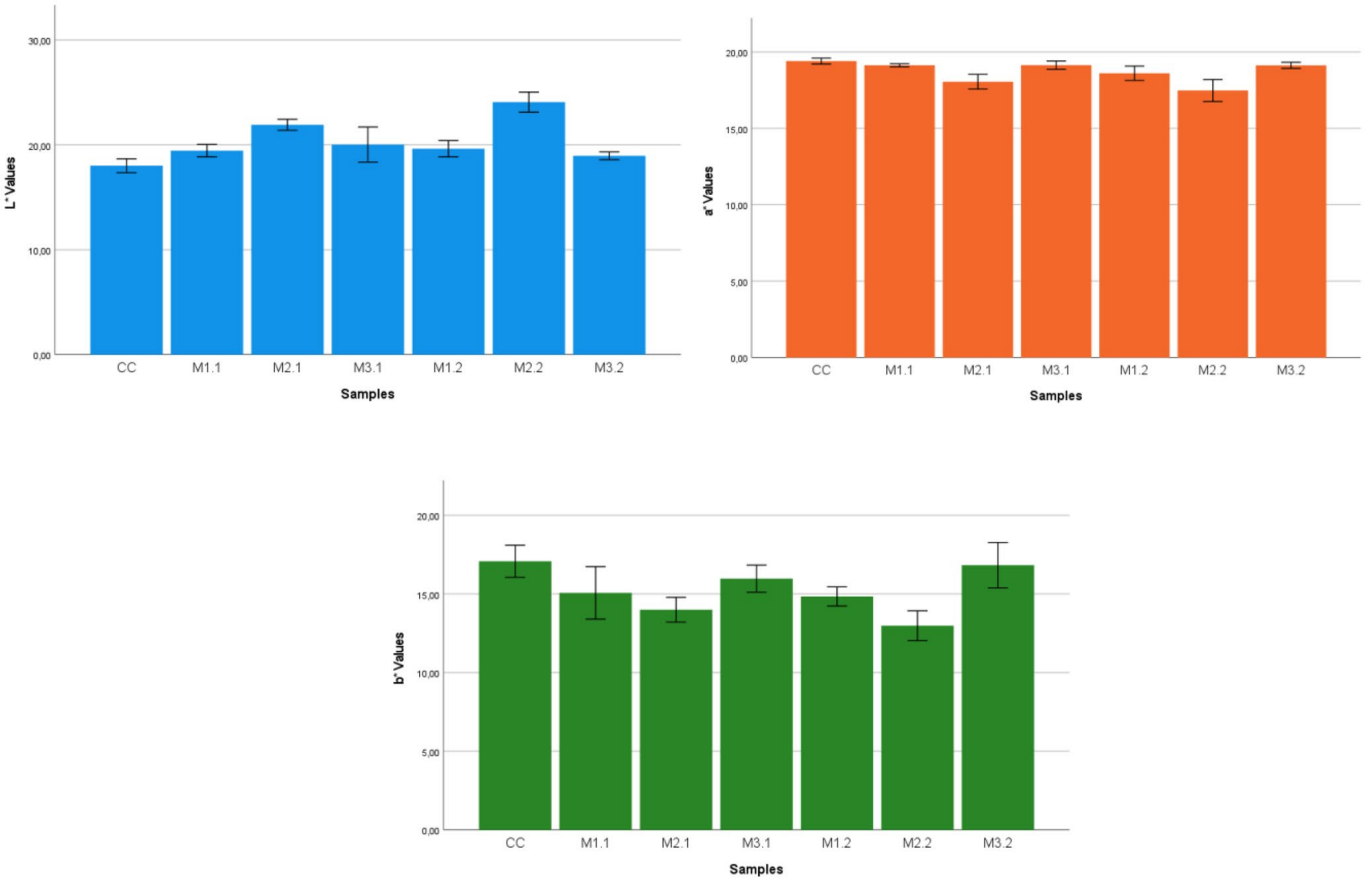
The L*, a*, and b* values of Kombucha samples. M1.1, 180 mT 1 h; M2.1, 240 mT 1 h; M3.1, 300 mT 1 h; M1.2, 180 mT 2 h; M2.2, 240 mT 2 h; M3.2, 300 mT 2 h.

The color values of the samples are shown in Table 5. Magnetic field application was significant in the color values of kombucha samples (*p* < 0.05). According to the results of the variation analysis, it was determined that magnetic field intensity and magnetic field intensity × time interactions were very highly significant (*p* < 0.0001) on the L* value, while magnetic field intensity × time interaction was very highly significant (*p* < 0.0001) on a* and b* values. Time interaction was highly significant (*p* < 0.01) on the L* value; magnetic field intensity and time interactions were highly significant (*p* < 0.01) on the a* value, while magnetic field intensity interaction was highly significant (*p* < 0.01) on the b* value. Changes in color values are given in Figure 2.

**TABLE 5:** Effects of applied magnetic field intensity time and magnetic field intensity × time interactions on the color values of the samples.

Samples	L*	a*	b*
Variation (*p* value)			
M	< 0.0001	0.008	0.008
T	0.004	0.006	0.163
M × T	< 0.0001	< 0.0001	< 0.0001
Correlation (*r*)			
M	0.194	0.007	0.034
T	0.268	−0.392	−0.223

*Note:* Statistical significance: *p* < 0.0001, very highly significant; *p* < 0.01, highly significant; *p* < 0.05, significant; *p* > 0.05, not significant.

Abbreviations: M, magnetic field intensity; T, time (hour).

L* value, an indicator of brightness in foods, increased with magnetic field application. The highest L* value was determined in the M2.2 sample. a* value decreased depending on the application. The lowest a* value was determined in the M2.1 sample. b* value started to decrease with magnetic field application but showed an increasing trend as the application time was prolonged. The hue of Kombucha may vary from pale yellow to dark reddish brown, depending on various factors affecting the fermentation process (van Wyk et al. 2023). The primary determinant of the color of Kombucha is the variety of tea used as a base. The fermentation time also plays a role, as longer fermentation times can result in a more intense and sour taste and a darker coloration (Wang et al. 2023). The application of the magnetic field influenced the count and variety of bacteria constituting SCOBY. It is thought that different metabolites, particularly organic acids, produced by altering fermentation duration and intensity influence the color shift of Kombucha tea.

**FIGURE 3:**
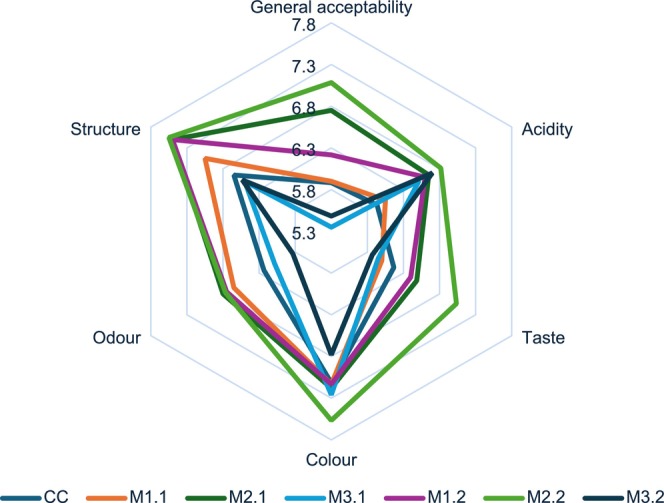
Sensory properties of kombucha tea samples.

The sensory evaluation of the samples is shown in Figure 3. The sensory evaluation revealed that participants found no significant differences in the color and structure values of the samples; however, notable changes were observed, particularly in the taste, smell, and flavor values (*p* < 0.05). General acceptability values exhibited significant differences among the samples (*p* < 0.05). The participants assigned the highest general appreciation scores to sample M2.2, followed by samples M2.1 and M1.2, in that order. Sample M3.2 received the lowest preference among the participants.

## 4 Conclusion

Kombucha is a traditional fermented tea that has been consumed since ancient times. In recent years, with the increasing health awareness of consumers, interest in fermented products containing probiotics and postbiotics has increased. This has made Kombucha tea even more popular. Magnetic field applications in foods generally aim to ensure food safety by microbial inactivation. Research indicates that the magnetic field's intensity and application duration can enhance the growth of microorganisms. This study subjected Kombucha culture SCOBY to varying intensities and durations of magnetic field exposure. The count of microorganisms affected by the magnetic field escalated. The elevated count of microorganisms influenced the fermentation process. At the end of the fermentation process, there was a notable increase in the concentrations of acetic acid, D‐glucuronic acid, gluconic acid, lactic acid, and citric acid. The rise in organic acid concentration corresponded with increased titration acidity of Kombucha tea, whereas the brix values declined. Concurrently, it was noted that there was an increase in the total concentration of phenolic compounds as well as in the capacity for antioxidant activity, as measured by DPPH assays.

D‐glucuronic acid enables the host to benefit more from antioxidants and thus increases the beneficial effects of antioxidants. After magnetic field application, Kombucha tea, which is already rich in this acid, has become even more prosperous in D‐glucuronic acid. Probiotics are live microorganisms that help the host's health when given sufficiently. Kombucha tea is an excellent source of natural probiotics. Postbiotics are substances produced by microorganisms during the fermentation process. Their presence in the environment consists of by‐products synthesized by other fermentative microbial flora, mainly lactic acid bacteria, and has beneficial effects on human health. Kombucha tea serves as a natural source of these chemicals. Applications of magnetic field intensity stimulate the development of probiotic microorganisms, while probiotics concurrently enhance the synthesis of postbiotic components. They also positively affect the sensory properties.

According to the study's findings, using SCOBY culture stressed with a magnetic field considerably improves Kombucha tea's functional qualities and nutritional value. Magnetic field application at various intensities and durations can boost the health benefits of Kombucha culture SCOBY.

## Author Contributions

**Azize Atik:** conceptualization (equal), investigation (equal), writing – review and editing (equal). **Gökhan Akarca:** formal analysis (equal), resources (equal), writing – original draft (equal). **Ayşe Janseli Denizkara:** data curation (equal), formal analysis (equal), investigation (equal).

## Ethics Statement

The authors have nothing to report.

## Consent

The corresponding author and all the co‐authors participated in the preparation of this manuscript.

## Conflicts of Interest

The authors declare no conflicts of interest.

## Data Availability

The original data with the respective analysis corresponding to the results shown in this work are available up to reasonable requirements.
